# Pyoderma gangrenosum associated to the use of cocaine/levamisole. Series of three cases and literature review^☆^^☆☆^

**DOI:** 10.1016/j.abd.2020.06.014

**Published:** 2021-02-06

**Authors:** Manuel Martínez-Gómez, Joan Andrés Ramírez-Ospina, Juan David Ruiz-Restrepo, Margarita María Velásquez-Lopera

**Affiliations:** Service of Dermatology, Department of Internal Medicine, Faculty of Medicine, University of Antioquia and San Vicente Fundación Hospital, Medellín, Colombia

**Keywords:** Cocaine, Levamisole, Pyoderma gangrenosum

## Abstract

Pyoderma gangrenosum associated to the use of cocaine/levamisole is a rare condition associated to their consumption. Cocaine use is frequent in Colombia, and the substance is contaminated with levamisole, an anthelmintic that increases the psychotropic effects and enhances its side effects. We present three clinical cases of patients with ulcerated lesions, in which the diagnosis was pyoderma gangrenosum secondary to the use of cocaine contaminated with levamisole. This called the attention of the health staff to investigate the abuse of substances in gangrenous pyoderma and also evidence that the interruption of consumption was the basis of management.

## Introduction

In 2016, 17 million people consumed cocaine, according to a report of the United Nations. In Colombia, it is estimated that cocaine is the second most consumed drug; the contamination with levamisole increases the psychotropic action and side effects. The medical use of levamisole was suspended in 1999 due to reports of agranulocytosis; currently, it is only available as an anthelmintic veterinary. It is believed that most cocaine is contaminated with this compound. It has been described that the combination of cocaine/levamisole produces vasculitis and serious vasculopathies; however, the onset of pyoderma gangrenosum (PG) has been seldom described. This study presents three PG cases associated with cocaine/levamisole use, a disease that must alert the medical staff to investigate the abuse of psychoactive substances as a part of the integral approach of those patients.

## Case report

### Patient 1

**Figure 1:**
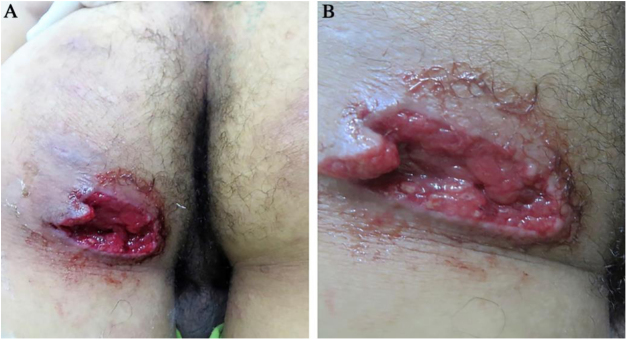
(A and B). Deep ulcer with irregular, well-defined borders, piercing, mild perilesional erythema, clean fundus, granular, without exudation on the left gluteus.

19-year-old male, presented to this service due to 5 days of papular erythematosus lesion on the left gluteus, which later became ulcerated, being extremely painful with seropurulent exudation, in addition to subcutaneous nodules on the palms, soles, elbows, and Achilles tendon (Fig. 1). The patient presented fever, arthralgia, and generalized myalgia. Medical history of spondylarthritis, oral ulcers and cocaine consumption for two years, last drug use one month prior to the consultation. At the physical exam, deep ulcer with well-defined irregular borders were observed, without erythema or inflammatory signs on the border, clean fundus, granular aspect, and no secretions. A skin biopsy was taken, where a clinical-pathological diagnosis of gangrenous pyoderma and vasculitis due to cocaine levamisole was reached; cultures were negative. Treatment was initiated with 60 mg prednisolone and 500 mg sulfasalazine every 12 hours, as well as toxicologic and psychiatric management of the addiction, with improvement

### Patient 2

**Figure 2:**
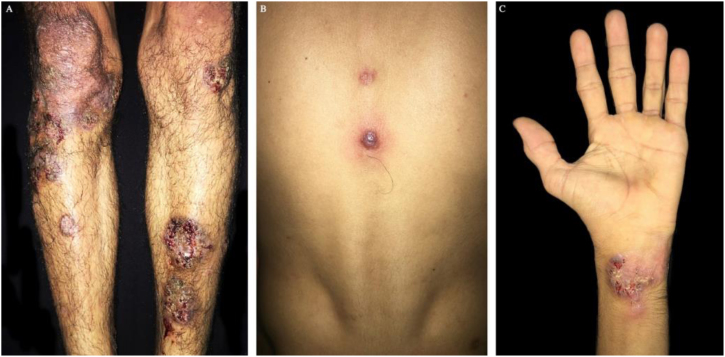
(A and C). Erythematosus nodules ulcers with violet brownish, well-defined, slightly raised borders, cribriform in the middle on legs and hands. (B) Violet papule with central vesiculation on the back dorsum.

**Figure 3:**
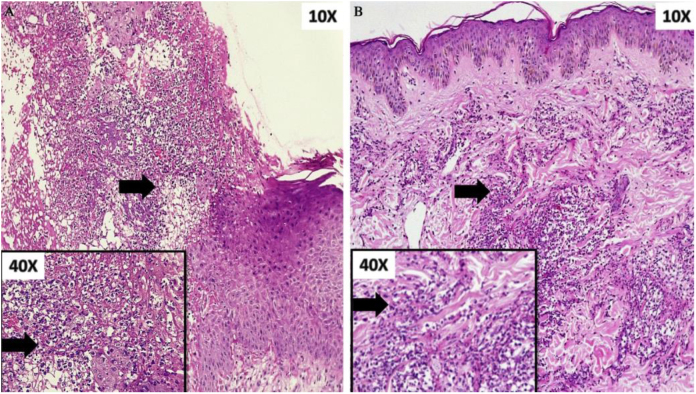
(A) Ulcer with abundant neutrophilic infiltrate on the papillary and reticular dermis. Box: detail of cellular component (arrow). (B) Dense perivascular inflammatory infiltrate of neutrophils. Box: detail of cellular component (arrow).

30-year-old male, presented to this service due to nodular erythematosus and painful lesion that later became ulcerated, which started one year prior, localized initially on the lower right limb and then with compromise of the upper limbs, ears, penis, and trunk (Fig. 2). Some of the lesions persisted, while others healed spontaneously. Ten days before hospital admission, the lesions grew in number and size. Medical history of pulmonar tuberculosis treated, use of tetrahidrocannabinol and cocaine for five years, last use 10 days prior to the consultation. At the physical exam, multiple erythematosus nodules and ulcers were observed, with defined violet-brownish borders, slightly raised and undermined, some others with more diffuse borders, center of cribriform aspect with bleeding spots and other regions with hypopigmented scars, localized prevailing on the lower limbs, some on the upper limbs. A skin biopsy was taken and a clinical-pathological diagnosis of vasculitis and pyoderma gangrenous due to cocaine and levamisole use was reached; cultures were negative (Fig. 3). Immunosuppressor management was not established, cocaine use was suspended, and this stopped the onset of new lesions. Toxicologic and psychiatric management of the addiction was continued, with improvement.

### Patient 3

**Figure 4:**
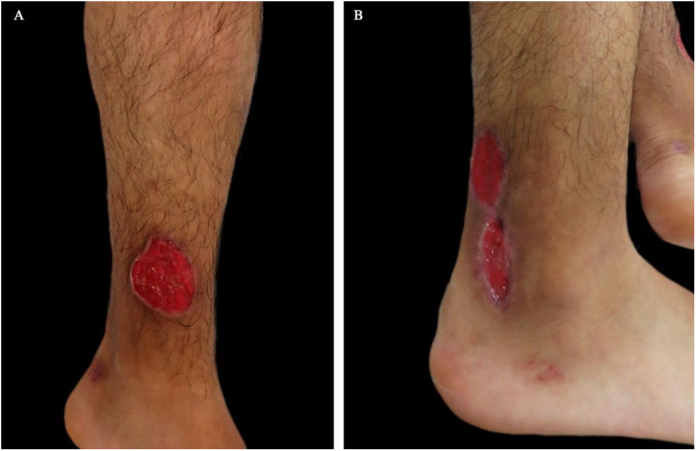
(A and B). Superficial ulcers, round in shape, well-defined, raised and slightly violet borders on both legs.

23-year-old male, who consulted due to the appearance of violet macule that afterwards got ulcerated on the right ankle and ipsilateral knee, of four months of evolution extremely painful, associated to asthenia, adinamia, and myalgia (Fig. 4). Then with the appearance of similar lesions on the contra-lateral lower limb, on both hands (dorsum) and on the ears, some of them scared over spontaneously. Moreover, with inflammatory polyarthralgia with compromise of shoulders, elbows, wrists, knees, and ankles. Medical history of alcoholism, use of tetrahydrocannabinol and cocaine, last use two weeks ago. To the physical examination with multiple superficial, ulcers, with well-defined borders, circular, raised, discreetly violet on lower limbs, with variable sizes, among 0.5 and 3 cm of diameter. Studies of extension were done, getting a diagnosis of vasculitis, PG, and nephrotic syndrome with dialytic urgency due to cocaine and levamisole; cultures were negative. Pulses of methylprednisolone 500 mg for three days were initiated, ambulatory 500 mg of cyclophosphamide a month and 50 mg of oral prednisolone. Toxicologic and psychiatric management of the addiction was continued, with improvement.

**Figure 5:**
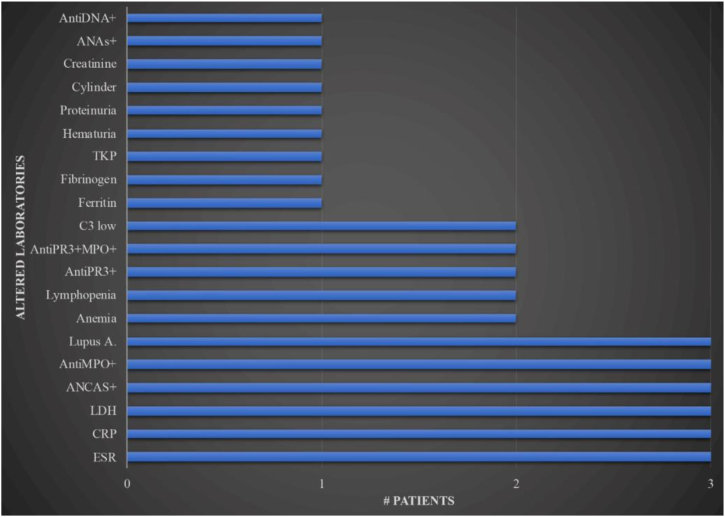
Laboratorial exams in patients with pyoderma gangrenosum related to cocaine. (X axis, number of patients; Y axis, altered paraclinical). ANAs, antinuclear antibodies; ANCAS, antineutrophil cytoplasmic antibodies, TKP, total kinase protein, AntiPR3, proteinase 3; AntiMPO, myeloperoxidase; Lupus A, lupus anticoagulant; LDH, lactic dehydrogenase; CRP, C reactive protein; ESR, erythrocyte sedimentation rate.

The laboratorial exams can be seen in Fig. 5.

## Discussion

PG associated with cocaine/levamisole is a recently described disease. Twenty-three cases published in the English-speaking literature were retrieved^2^, ^3^, ^4^, ^5^, ^6^, ^7^, ^8^, ^9^, ^9^, ^11^ (Table 1), none of them in Colombia. Unlike the classic form, PG associated with cocaine is accompanied by vasculopathy and cutaneous or systemic vasculitis. Antibodies, especially ANCAS and the positive lupus anticoagulant is exacerbated with the use of cocaine, it gets better with abstinence and it has a fast response to immunosuppressants. The combination of cocaine/levamisole triggers a cascade of immunological events that cause death of neutrophils, with the formation of extracellular networks and the exposure to antigens.

**Table 1:** Cases of pyoderma gangrenosum associated with cocaine or cocaine/levamisole use reported in the literature.

Nº	Authors	Age and sex	Time of consume	Time to onset PG	Localization	Rechallenge^a^	Vasculitis	Systemic symptoms	Autoantibodies	Treatment	Cocaine level
1	Friedman et al. (2005)^1^	27 F	NR	NR	Face, legs, arms and back. Septum destruction, ethmoidal and maxillary sinusitis	Yes	No	No	ANAs+ 1:640, p-ANCAS + against PR3	NR	Yes
2	E. Roche et al. (2008)^2^	30 M	2 years	3 months	Initially back, later trunk and limbs	Yes	No	No	No	Cyclosporine, topical methotrexate, tacrolimus, and infliximab after interrupting cocaine consumption	Yes
3	E. Roche et al. (2008)^2^	37 M	10 years	4 months	Back, upper third of the arms, and cheek	Yes	Yes	No	No	Infliximab, topical tacrolimus, associated with cocaine abstinence	No
4	Camilla Bezerra da Cruz Maia et al. (2012)^3^	27 F	10 years	5 years	Left half-face and inferior members. Hard palate and nasal septum destruction	Yes	No	No	No	Azathioprine and prednisone resulted satisfactory with partial healing of the face	No
5	D. Jimenez-Gallo et al. (2013)^4^	54 F	5 years	2 months	Both legs. Saddlenose nasal deformity associated with oronasal fistula	Yes	No	Retiform purpura and lung involvement	p-ANCAS+ 1:80 for elastase and ANAs 1:40	Cyclophosphamide bolus	Yes
6	Phillip J. Keith et al. (2014)^5^	51 F	NR	2 months	Face, abdomen, back, thigh and pubis	Yes	No	No	P-ANCA+ > 1:640, ANAs+, AL+, ACL+	Prednisone and cocaine abstinence	No
7	Haneol S. Jeong et al. (2015)^6^	n = 8; ages and sex NR	NR	1 week to 4 weeks (median: 1 week)	Lower limbs (n = 8), upper limbs (n = 6), trunk (n = 3), face (n = 3)	Yes	n = 5	Retiform purpura (n = 3). Artrhalgias (n = 1)	ACL+ (n = 5), AL+ (n = 3), B2GP+ (n = 4) p-ANCA+ (n = 7), anti PR3+ (n = 4) y anti-MPO+ (n = 7), ANAs+ (n = 3) and FR+ (n = 1)	Prednisone were administered in 6 of 8 patients. Gentle wound care and cocaine avoidance in all patients with improvement	Yes
8	Carola Baliu-Piqué et al. (2016)^9^	40 F	NR	2 weeks	Breast, hips, upper and lower extremities	Yes	No	No	No	Methylprednisolone bolus, cyclosporine, infliximab, and mycophenolic acid	Yes
9	Ricardo Ruiz-Villaverde et al. (2016)^7^	38 M	NR	NR	Armpits, chest, pubis, and lumbar region	Yes	No	No	No	Prednisone	Yes
10	Rahul Sehgal et al. (2017)^8^	53 F	NR	6 months	On the upper back. Ulceration of bilateral nasal passages and nasal septum perforation	Yes	No	Right-sided multifocal pneumonitis and mild reactive lymphadenopathy.	No	Intralesional triamcinolone, local wound care, prednisone, dapsone, topical tacrolimus, cyclosporine, and cocaine discontinuation, resulting in gradual improvement	Yes
11	Ester Moreno-Artero et al. (2018) ^10^	37 M	NR	4 years	Cheeks and right gluteal area	Yes	No	No	c-ANCAS + against PR3 and AL+	NR	Yes
12	Ester Moreno-Artero et al. (2018) ^10^	34 F	NR	20 days	Both hands, lower back, and lower limbs	Yes	No	No	p-ANCAs + against elastase	NR	Yes
13	Ester Moreno-Artero et al. (2018) ^10^	43 M	3 years	6 months	Face, trunk, and lower limbs	Yes	No	No	ANCAs + against elastase	NR	Yes
14	Andrea Estébaneza et al. (2020)^11^	40 F	NR	6 months	Back, chin, and retroauricular area, destruction of the nasal septum and the lateral maxillary sinus wall	Yes	No	Left posterior perirenal and pararenal esteril abscess	No	Corticosteroids and cessation of cocaine use through a rehabilitation program, skin lesions and kidney abscess resolved.	No
15	Andrea Estébaneza et al. (2020) ^11^	51 M	NR	NR	Right Achilles tendon lesion	Yes	Yes	No	No	Prednisone and cocaine consumption were temporarily interrupted	Yes
16	Andrea Estébaneza et al. (2020) ^11^	54 M	NR	2 weeks	Back	Yes	No	No	ANAs+ (title 1/320).	Corticosteroid	Yes

^a^Recurred with repeat cocaine use.

F, female; M, male; NR, not registered; ANA, antinuclear antibody; p-ANCA, perinuclear anti-neutrophil cytoplasmic antibodies; PR3, Antiproteinase-3 antibody; AL, lupus anticoagulant; ACL, cardiolipin antibody; B2GP, beta-2 glycoprotein; anti-MPO, antimyeloperoxidase; FR, rheumatoid factor; c-ANCA, cytoplasmic antineutrophil cytoplasmic antibody.

In this series of cases, besides the cutaneous compromise, one of them presented a kidney failure that required dialysis. While Case 2 did not present compromise of other organs, systemic inflammation, diminished complement, and autoantibodies were observed. It is possible that factors of individual susceptibility, combined with others such as time, use frequency, and degree of cocaine contamination, might be involved in the type of clinical manifestation and its severity. The use of cocaine sets out a problem due to the cutaneous and systemic lesions, and their possible consequences, such as chronic kidney failure and even death.

In conclusion, three cases of PG associated with the use of cocaine/levamisole were presented, all of which presenting cutaneous compromise and one, acute kidney failure. Cocaine use suspension is the cornerstone of the treatment; therefore, these patients require a multidisciplinary management that includes specialists in addiction rehabilitation. These cases coincide with those described in the literature, which is why it was presumed that the cocaine used is contaminated with levamisole, as most of those commercialized. The dermatologist must be alert to these types of reactions associated with psychotropics; furthermore, it is important to inform the community of those additional risks related to the use of cocaine.

## Financial support

None declared.

## Authors' contributions

Manuel Antonio Martínez-Gómez: Study conception and planning; preparation and writing of the manuscript; data collection, analysis, and interpretation; Intellectual participation in propaedeutic and/or therapeutic management of studied cases; critical literature review.

Joan Andrés Ramirez Ospina: Study conception and planning; preparation and writing of the manuscript; data collection, analysis, and interpretation; intellectual participation in propaedeutic and/or therapeutic management of studied cases; critical literature review.

Juan David Ruiz-Restrepo: Approval of the final version of the manuscript; effective participation in research orientation; intellectual participation in propaedeutic and/or therapeutic management of studied cases; manuscript critical review.

Margarita María Velásquez Lopera: Approval of the final version of the manuscript; effective participation in research orientation; intellectual participation in propaedeutic and/or therapeutic management of studied cases; manuscript critical review.

## Conflicts of interest

None declared.
